# Innate immune dysregulation in celiac disease and refractory celiac disease: mechanisms, management and emerging therapies

**DOI:** 10.3389/fimmu.2026.1926029

**Published:** 2026-09-15

**Authors:** Sara Shaikh, Preeti Jain

**Affiliations:** Department of Life Sciences, Somaiya School of Basic and Applied Sciences, Somaiya Vidyavihar University, Mumbai, India

**Keywords:** autoimmune disorder, barrier dysfunction, celiac disease, innate immune dysregulation, interleukin-15, refractory celiac disease

## Abstract

Celiac disease is traditionally regarded as T-cell mediated autoimmune disorder driven by gluten-specific adaptive immune responses. In this review, we synthesise recent data showing how epithelial stress, IL-15 driven innate circuits and intraepithelial lymphocyte reprogramming contribute to both classical and refractory disease. It affects 1-3% of people worldwide, caused by gluten intake in individuals carrying HLA-DQ2/DQ8 haplotypes. Gliadin peptides such as 33-mer and p31-43, resist digestion, trigger zonulin release through CXCR3, disrupt epithelial barrier and induce an innate immune response through oxidative stress, EGFR signalling, and trans presentation of IL-15. In refractory celiac disease, this reprograms intraepithelial lymphocytes into cytotoxic, NK-like effectors expressing NKG2D and NKp30. These target MICA/B stressed epithelium independently of adaptive immunity, maintaining villous atrophy despite a strict gluten-free diet. Type I RCD shows polyclonal surface CD3+/CD8+ IELs whereas type II shows clonal aberrant IELs lacking surface CD3 and hypersensitive to IL-15 due to JAK/STAT mutations leading to enteropathy-associated T-cell lymphoma. This circuit is strengthened by gut dysbiosis, viral triggers and epigenetic changes. Emerging therapies including budesonide, cladribine, IL-15/JAK inhibitors, nutraceuticals and microbiota modulation are assessed against this framework. The manuscript proposes viewing RCD as a failure of innate immune regulation rather than solely as an adaptive response.

## 1 Celiac disease: an autoimmune disorder

Celiac disease is one of the most common chronic autoimmune inflammatory disorders of the small intestine (1). It is prevalent in many industrialised countries, affecting approximately 1-3% of the global population (2). Although the disease can occur at any age, the highest incidence of seroconversion is observed between 12 and 36 months of age. First-degree and, to a lesser extent, second-degree relatives have an increased risk for celiac disease (3).

According to WGO (World gastroenterology Organization) the incidence of celiac disease has risen over the last 20 years. Globally, its prevalence varies between approximately 1:100 and 1:300 individuals (4). The observed increase is partially due to improved diagnosis and partially a likely increase in disease incidence. The wider use of anti-tissue transglutaminase serology, active case finding and recognition of non-classical presentations have contributed to improved detection. The analysis of archived sera by independent longitudinal cohorts indicates an increase in seroprevalence, including approximately fourfold rise compared with sera collected between 1948 and 1954 (5, 6). Recent meta-analysis estimate global pooled seroprevalence at approximately 1.4% and biopsy at approximately 0.7%, with incidence continuing to rise in several populations (7, 8).

Several environmental drivers have also been proposed. Early-life viral infection such as reovirus infection, shown to disrupt oral tolerance to dietary antigen and rotavirus infection associated with increased risk (9, 10). Alterations in the early life microbiome, perinatal antibiotic exposure and caesarean delivery show consistent associations but no established role. Two independent randomised controlled trials demonstrated that the timing of gluten introduction does not alters disease risk in genetically susceptible infants (11, 12). Similarly, the frequent assertion that modern wheat breeding has increased gluten content is not supported by compositional analysis of historical cultivators (13), although the quantity of gluten consumed in early childhood does appear to influence risk (14). This divergence between epidemiological association and experimental confirmation is a recurring feature of the field and warrants caution in attributing the incidence rise to any single factor.

Celiac disease develops after dietary exposure to gluten in genetically predisposed individuals. These individuals are the carriers of HLA-DQ2 or HLA-DQ8 haplotypes located on chromosome 6, which encode specific major histocompatibility complex (MHC) class II human leukocyte antigen (15, 16). Although these haplotypes are present in approximately 30-40% of the population, only 1% out of them develop celiac disease. This suggests that the presence of HLA genes is necessary but not sufficient by itself to cause disease (1, 15, 17, 18).

Gluten comprises two major protein fractions: gliadins and glutenins. Gliadins are alcohol soluble, whereas glutenins are water and alcohol insoluble and characterised by a high content of glutamine and proline residues, which contribute to their antigenic properties, including the ability to bind to HLA molecules (15). Gliadins are further classified into sulphur-rich (α, β and γ) and sulphur-poor (ω) fractions. These gliadins contain immunogenic epitopes capable of activating immune responses in susceptible individuals (19, 20). In healthy individuals, complete degradation of gliadins into single amino acids renders them non-toxic. However, incomplete digestion by pepsin and trypsin generates toxic peptide fragments, particularly 33-mer(p55-87) and 25-mer (p31-55) (16). In susceptible individuals, these peptides can remain intact whilst passing through the intestine, accumulate and contribute to intestinal inflammation (2, 21–23).

These glutamine-rich peptides bind to the CXCR3 receptor present on intestinal epithelial cells, triggering the release of zonulin and leading to increased intestinal permeability. The increased permeability allows gliadin peptides to enter the lamina propria, where they are deamidated by tissue transglutaminase 2 (tTG2), resulting in a stronger binding affinity for HLA-DQ2 and DQ8 (19, 24). This eventually leads to the activation of innate and adaptive immune responses, causing chronic inflammation and tissue damage in the small intestine (19, 25). In addition to genetic predisposition and gluten exposure, environmental factors such as gut microbiota composition, timing and manner of gluten introduction, viral infection and alteration in innate immune cell populations may also influence disease onset and progression by altering gluten metabolism and immune responses (26–30). Immune responses to specific gliadin peptides, involving both adaptive and innate immune mechanisms, play a central role in this inflammatory process (23, 31, 32).

The understanding of CD pathogenesis has changed considerably over past decade. The gluten specific CD4+ T-cell response has been extensively characterised and remains the defining event of the disease (15). However, this adaptive model could not explain that why villous atrophy requires cytotoxic IEL activity and why a small proportion of patients continue to show mucosal damage even after GFD. The demonstration that IL-15 reprogrammes IELs for TCR independent killing establish that an innate immune circuit operates alongside the adaptive and can become independent of it (33, 34). This review is organised around three questions that follow from this shift: which epithelial signals sustain IEL activation once antigen is removed, why IL-15 blockade has produced only partial clinical benefit, and which features distinguish patients who progress to RCD II.

Prospective birth cohort studies have traced the development of celiac disease over time and the data shows that from genetic risk to early warning signs like unusual lipid patterns and microRNA changes before gluten is even introduced. The disease then progresses to a stage characterised by the production of anti-tissue transglutaminase (anti-tTG) antibodies, which contributes to damage of small intestinal villi, resulting in villous atrophy (29, 30).

The clinical presentation of CD varies widely, ranging from asymptomatic or minimally symptomatic disease to severe malabsorption. Whilst some patients present with classic gastrointestinal symptoms such as chronic diarrhoea, weight loss, vomiting, and nutrient deficiencies, many exhibit non-classical or extraintestinal manifestations, including anaemia, osteoporosis and neurological symptoms (3). Based on clinical presentation and disease progression, celiac disease is broadly classified into typical, atypical, asymptomatic, potential and refractory forms. The typical form is characterised by malabsorptive gastrointestinal symptoms, whereas the atypical CD presents mainly with extra-intestinal manifestations with few or no gastrointestinal symptoms. Asymptomatic CD is defined by positive serological and histological findings in the absence of clinical symptoms, whilst potential CD refers to genetically susceptible individuals with positive serology but normal or mildly abnormal intestinal histology, indicating a risk of developing celiac disease in the future (16, 35). Refractory celiac disease (RCD) is a rare but severe form characterised by persistent malabsorptive symptoms and villous atrophy despite adherence to a strict gluten-free diet (1, 4, 34). Typical features include chronic diarrhoea, weight loss, anaemia and nutritional deficiencies (36). Approximately 5-30% of patients fail to achieve complete clinical and histological remission or experience relapse despite an initial response to a gluten-free diet. RCD is classified into two subtypes: type 1, with normal intraepithelial lymphocytes (IELs) and type 2, characterised by aberrant intraepithelial lymphocytes (IELs) (1, 4, 37). The RCD is uncommon, affecting about 0.3-1.5% of CD patients, and occurs more frequently in women (38).

A strict gluten free diet (GFD) remains the only established therapy for CD and typically results in clinical improvement and mucosal healing. However, such diets may be nutritionally imbalanced, leading to deficiencies of vitamin B12, folate and vitamin D, reduced fibre intake and metabolic complications such as weight gain, dyslipidaemia and non-alcoholic fatty liver disease, partly due to high glycaemic index and altered macronutrient composition of many processed gluten-free products (3).

### 1.1 Literature methodology

This narrative review is based on a structured literature search of PubMed, Scopus and Web of Science, covering publications from year 2000 to 2026, supplemented by earlier seminal papers identified through citation tracking where they established foundational concepts. Search terms combined the following in Boolean manner: (‘celiac disease’ OR ‘coeliac disease’ OR ‘refractory celiac disease’ OR ‘RCD’) AND (‘innate immunity’ OR ‘ interleukin-15’ OR ‘IL-15’ OR ‘intraepithelial lymphocyte’ OR ‘IEL’ OR ‘NKG2D’ OR ‘epithelial barrier’ OR ‘gut microbiota’ OR ‘enteropathy associated T-cell lymphoma’). Inclusion criteria were: peer-reviewed original research, systematic reviews and clinical guidelines published in English; studies addressing mechanisms of innate immune activation, IEL biology, or therapeutic interventions in CD or RCD. Exclusion criteria were: conference abstracts without full text publication, articles addressing non celiac gluten sensitivity. Reference lists of included articles were screened for additional relevant sources.

## 2 Refractory celiac disease: mechanistic insights

The classical model of celiac disease pathogenesis emphasises the role of the adaptive immune system in recognising gluten-derived peptides, leading to villous atrophy, crypt hyperplasia and malabsorption (15). Strict adherence to a GFD results in clinical and histological remission in the majority of the patients, confirming the central role of gluten as the initiating antigen (39). However, Despite strict dietary adherence and exclusion of alternative causes of enteropathy, approximately 0.3-0.4% of patients develop persistent malabsorption and villous atrophy (3, 27, 40). These individuals are classified as having RCD, a condition associated with persistent mucosal injury, increased morbidity and a very high risk of enteropathy-associated T-cell lymphoma (EATL), particularly in RCD type II (34, 40). The difference between the CD and RCD is represented in **Figure 1**.

**Figure 1:**
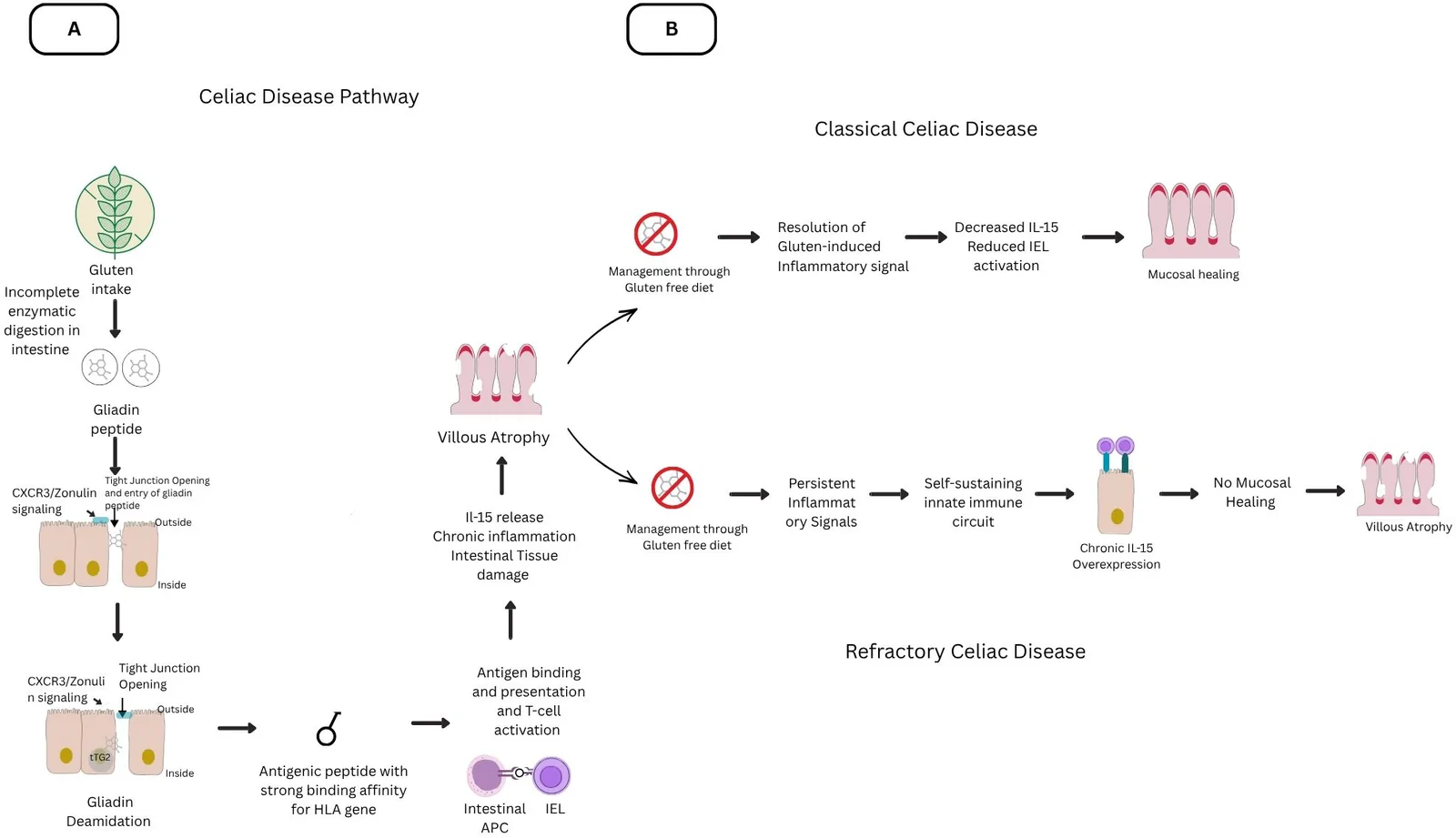
Classical celiac disease vs. refractory celiac disease. **(A)** Mechanistic insights of Celiac Disease. **(B)** Differential operating pathway in Classical VS Refractory Celiac Disease. tTG2, Tissue Transglutaminase 2; IEL, Intraepithelial lymphocytes; ROS, Reactive oxygen species.

RCD challenges the conventional gluten-centric adaptive immune model. Persistent villous atrophy in the absence of ongoing antigen exposure suggests the presence of self-sustaining immune circuits within the intestinal epithelium. Evidence indicates that innate immune dysregulation, driven by epithelial stress responses, chronic interleukin-15 (IL-15) signalling, and pathological reprogramming of IELs plays an important role in this process (15). Furthermore, gliadin-induced endocytic alteration and IL-15 trans presentation contribute to epithelial vesicular trafficking defects and chronic overexpression of surface IL-15. This promotes IEL dysfunction and crypt hyperplasia, ultimately leading to the development of an antigen-independent innate immune circuit representing refractory CD (41).

### 2.1 Intestinal epithelial innate immune responses in RCD

The intestinal epithelium functions not only as a physical barrier but also as a highly specialised innate immune interface capable of sensing dietary antigens, microbial products and cellular stress signals (42, 43). Enterocytes express a broad range of pattern recognition receptors (PRRs), stress response pathways and cytokine signalling machinery that enable rapid communication with immune cells, particularly IELs (43, 44).

Of the gliadin peptides that reach epithelium, the p31–43 peptide primarily activates innate immune pathways independent of T-cell recognition (22, 41, 45). Gliadin binding to the CXCR3 receptor on enterocytes induces zonulin release by recruiting MyD88, leading to tight junction disassembly and increased intestinal permeability (46, 47). This barrier dysfunction facilitates the translocation of immunogenic peptides into the lamina propia and simultaneously generates an intracellular stress signal within epithelial cells (47, 48). However, this model does not account for all gliadin translocation. CXCR3 independent routes of gliadin translocation also exist, including secretory IgA mediated retrotranscytosis through the transferrin receptor CD71. Barrier dysfunction in CD is therefore likely to be multifactorial rather than attributed to a single receptor (49). *In vitro* studies using human epithelial Caco-2 cell have further confirmed this. One study shows that pepsin-trypsin-digested gliadin induces epithelial barrier disruption and inflammatory responses (2, 50). Additionally, an ex vivo study employing intestinal explant system demonstrated that gliadin exposure led to a significant increase in permeability across all explant groups compared with media controls; however, the effect was most pronounced in samples from patients with active CD, whilst biopsies from patients in remission exhibited a more attenuated response (51).

After endocytic uptake, gliadin peptides accumulate within endosomal and lysosomal compartments, where the p 31–43 fragments disrupt endosomal maturation and vesicular trafficking. The peptide accumulates in the early endosome and delays its maturation into late endosomes. This results in prolonged accumulation of epidermal growth factor receptor (EGFR) and IL-15/IL-15Rα complex and increases their recycling back to the plasma membrane (41). This alteration prolongs (EGFR) signalling, promotes crypt hyperplasia, and establishes a persistent epithelial stress state (21, 45). In parallel, p31–43 activates innate like pathways through Toll-like receptor 7 (TLR7) and IFN α associated signalling, linking gliadin exposure directly to innate immune activation independent of adaptive immunity (12, 30, 34).

Persistent epithelial stress is further amplified by mitochondrial dysfunction and oxidative stress. Gliadin exposure induces excessive reactive oxygen species (ROS) production, impaired mitochondrial dehydrogenase activity and mitochondrial DNA damage, resulting in chronic redox dysregulation within enterocytes (25, 52, 53). An *in vitro* study shows that when the Caco-2 monolayer is treated with gliadin, it causes oxidative damage, which causes physical changes such as cell proliferation, cytoskeleton rearrangement and disruption of tight junction integrity (50, 54). This oxidative environment activates NF-κB signalling and establishes a self-amplifying cycle of oxidative and inflammatory stress. Although epithelial cells initiate compensatory mitochondrial biogenesis and antioxidant responses, sustained oxidative stress contributes to chronic epithelial dysfunction and continuous activation of innate inflammatory pathways (25, 52, 53).

The stressed epithelial phenotype is also characterised by endoplasmic reticulum stress and increased expression of non-classic MHC molecules and stress ligands such as MICA and HLA-E. These molecules act as danger signals that directly activate IELs in an antigen-independent manner. Consequently, IELs acquire cytotoxic and natural killer (NK) like properties capable of inducing epithelial injury through perforin and granzyme mediated mechanisms. This process highlights how epithelial stress itself can sustain mucosal inflammation even in the absence of persistent adaptive immune stimulation (48)

Amongst the innate cytokines involved, interleukin-15 (IL-15) is considered a central regulator of epithelial-IEL crosstalk in CD and RCD. Under normal physiological conditions, IL-15 maintains epithelial integrity and IEL homeostasis; however, in CD its expression becomes markedly upregulated and chronically trans-presented by epithelial cells through IL-15 receptor alpha (IL-15Rα) (55–57). Elevated IL-15 lowers the activation threshold for IELs, enabling cytotoxic activation independent of T-cell receptor (TCR) signalling. This cytokine-driven reprogramming shifts IELs towards a persistent innate effector phenotype that contributes directly to epithelial destruction (56, 58). Although IL-15 dominates the innate immune circuit, it does not act alone. IL-21, produced by lamina propria CD4+ T cells, acts together with IL-15 to amplify IEL cytotoxicity and IFNϒ production (59). IL-15 also impairs Smad3 dependent TGF- β signalling in intestinal lymphocytes through sustained activation of c-Jun N-terminal kinase, which removes an important regulatory brake on the intraepithelial compartment. Notably, neutralising IL-15 restored TGF- β dependent transcription in biopsies from patient with active disease, which indicates that this defect is reversible (60, 61). The relative contribution of these interacting cytokine pathways to RCD is not well defined. Disrupted vesicular trafficking further intensifies the process. The P31–43 peptides delay early endocytic maturation, resulting in recycling and prolonged surface accumulation of IL-15/IL-15Rα complex without requiring new protein synthesis. Sustained IL-15 trans-presentation maintains chronic IEL activation and epithelial stress signalling (41). In addition, cooperative signalling between EGFR and IL-15Rα activates ERK1/2 and STAT5 pathways, promoting crypt enterocyte proliferation and reciprocal amplification of IL-15 and EGF signalling (62). Together, these mechanisms establish a self-perpetuating innate immune circuit within the intestinal epithelium. Epithelial stress increases surface expression of MICA/B and HLA-E. This sustains the trans presentation of IL-15 which in turn lowers the activation threshold of IELs and permits NKG2D mediated killing of enterocytes without TCR engagement. The resulting epithelial injury reinforces the stress response, so the circuit is maintained without further gluten exposure once it is established (22, 41, 45) (**Figure 2**). Animal models of celiac disease also reveal that the condition requires multiple converging factors to fully develop. These include an HLA-DQ2/DQ8 restricted T cell response to gluten, an innate immune reaction involving IL-15 overexpression and zonulin-mediated gut permeability and an autoimmune component with anti-tissue transglutaminase antibodies. IL-15 transgenic mice expressing HLA-DQ8 show dramatic expansion of cytotoxic intraepithelial lymphocytes and inflammation, yet they lack villous atrophy. This confirms that epithelial IL-15 is sufficient to drives immune dysregulation independently of gluten, but not enough to produce histological lesions that define the disease (63). The circuit described above should therefore be understood as necessary for persistence rather than as a complete account of tissue damage.

**Figure 2:**
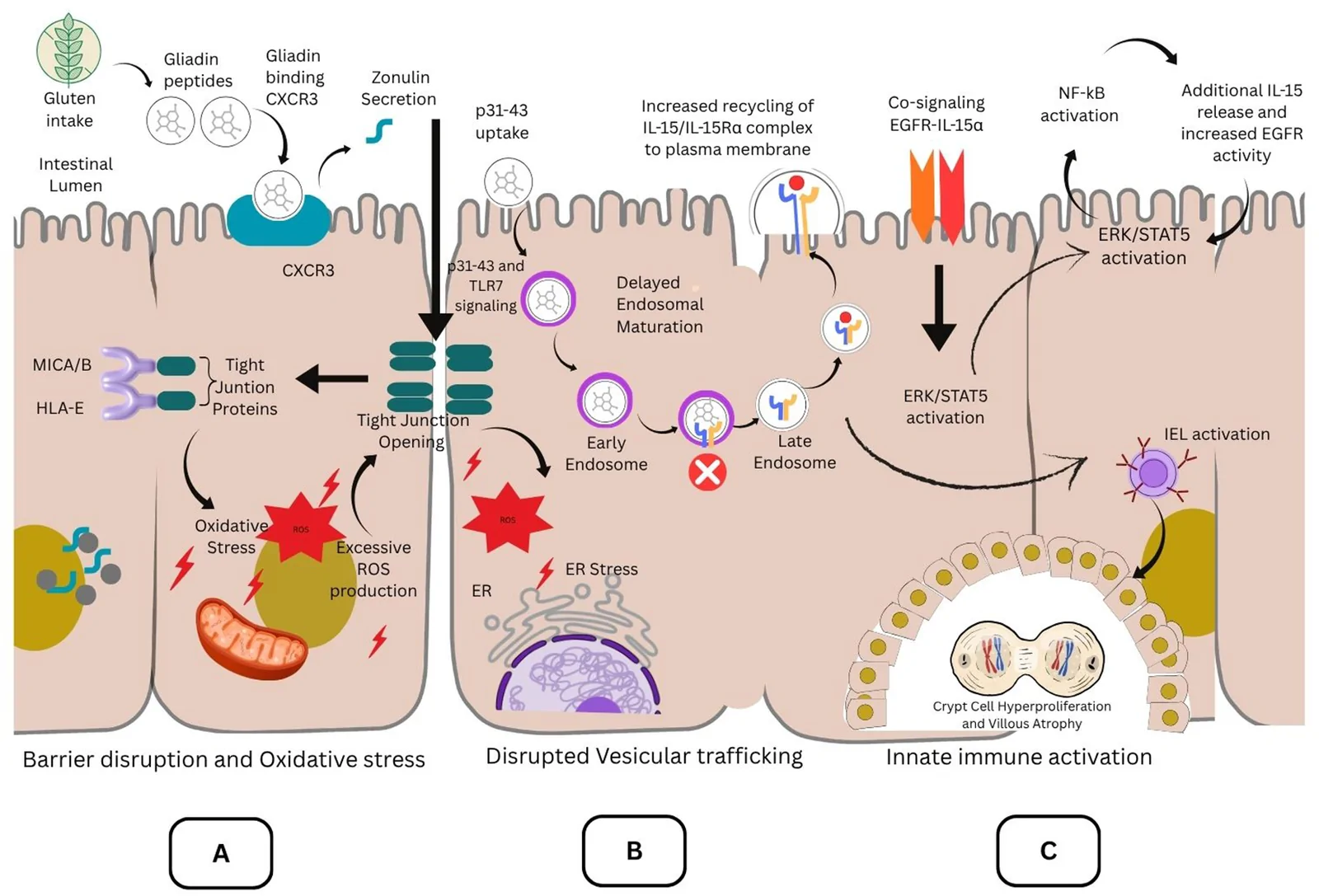
Innate immune responses in refractory celiac disease. **(A)** Barrier disruption and oxidative stress after gluten intake. **(B)** Disrupted Vesicular Trafficking. **(C)** Innate immune activation and Villous Atrophy. TLR7, Toll like receptors; EGFR, Epidermal growth factor receptor; IEL, Intraepithelial lymphocytes.

### 2.2 IEL reprogramming: central player in RCD

IEL is the important part of the IL-15 circuit discussed above, and its reprogramming converts a reversible epithelial stress response into sustained tissue damage. In the healthy individuals, IELs comprise a heterogeneous population of immune cells embedded within the intestinal epithelium, including conventional αβ T cells, γδ T, and Innate-like lymphocytes with NK cell features. It contributes to immune surveillance, epithelial repair and tolerance with cytotoxic activity tightly regulated by inhibitory receptors and antigen specificity (15). αβ T cells are the main cytotoxic effectors and undergoes reprogramming which is further described in the section. γδ T-cells are also expanded in celiac disease. Unlike the αβ, their elevation persists after mucosal healing on GFD which makes them a durable marker of previous disease. Chronic inflammation permanently reshapes these cells, with loss of naturally occurring CD8 αα+ TCRγδ subset that is not restored by gluten withdrawal (64).

Recent immunophenotyping has expanded the characterisation of intestinal IELs beyond activating receptors. LAIR1 is an ITIM bearing inhibitory receptor expressed by normal small intestinal IELs along with CD3, CD8, CD103 and TCRβ. In celiac disease, LAIR1 positive IELs and lamina propria immune cell are increased. Transcriptomics analysis also demonstrates activation of LAIR1 pathway and increased expression of other inhibitory molecules such as BTLA. The celiac lamina propria is additionally enriched in CD163+ macrophages and cells expressing PD-L1, CD103 and CD56 (65) Ex vivo transcriptomic data from human duodenal tissue has shown coordinated changes in genes involved in immune signalling. Through the integration of differential gene expression analysis with protein–protein interaction network mapping, distinct clusters of both upregulated and downregulated genes associated with immune signalling pathways, cell cycle regulation, and absorptive and metabolic functions were identified. This systems biology approach provides insight into the molecular signatures underlying intestinal inflammation and tissue injury in celiac disease (66).

In refractory celiac disease, persistent epithelial IL-15 signalling induces significant phenotypic and functional alterations in IELs (67–69). Cytotoxic lymphocytes progressively lose dependence on T-cell receptor (TCR)-mediated antigen recognition and acquire NK-like characteristics, including increased expression of activating receptors such as NKG2D, CD94 and NKp30, along with reduced inhibitory receptor expression (33, 67, 70, 71). In parallel, IL-15 promotes upregulation of FAS/Fas ligand and stress ligand such as MICA on intestinal epithelial cells. Interaction of NKG2D on IELs with MICA-expressing enterocytes generates an antigen-independent activation signal that promotes epithelial cell killing (32, 33, 72). This reprogramming shifts IELs recognition from peptide-MHC complexes towards stress-induced ligands, resulting in epithelial damage and crypt cell hyperproliferation independent of direct gluten recognition (33, 73).

IL-15 also induces resistance to activation-induced cell death, enabling accumulation of highly cytotoxic IEL populations (33, 74). These reprogrammed IELs mediate epithelial injury through perforin and granzyme-dependent cytotoxicity triggered by recognition of stress ligands such as MICA A/B (33, 45). In untreated CD mucosa, MICA/B expression is observed in enterocytes and IELs populations, reflecting epithelial stress and inflammation. Although expression decreases following a GFD, persistence of MICA/B+ T cells suggests ongoing innate immune dysregulation. In this environment, NKG2D-mediated activation of NK cells and IELs sustains cytotoxicity against stressed enterocytes independently of adaptive signals, contributing to persistent mucosal damage (32, 48, 69, 75).

Chronic gluten-driven inflammation further remodels the intraepithelial immune niche by increasing expression of activating receptors such as NKG2D, NKp30 and NKG2C on T cells, NK cells and NKT cells, enabling recognition of MICA/B and HLA-E on enterocytes and promoting epithelial lysis even in the absence of classic TCR recognition (28). In most patients, withdrawal of gluten leads to normalisation of epithelial stress responses, reduced IL-15 expression and restoration of IEL homeostasis (76). However, in RCD, this resolution fails to occur, resulting in persistent cytokine expression and continued display of stress ligand, leading to ongoing mucosal damage despite dietary adherence (27, 68).

RCD represents a progressive failure of innate immune regulation within the epithelial niche. RCD type I is characterised by polyclonal IELs population retaining normal phenotype, with surface markers, including CD3, CD8 and TCR expression preserved. In contrast, RCD II is characterised by clonal expansion of aberrant IELs that lack surface CD3, CD8 and TCR whilst retaining cytoplasmic CD3 (27, 36, 46, 68). The relationship between the two subtypes is not fully understood. It remains unresolved whether the aberrant clone in type II arises from a distinct innate like T-IEL precursor present before disease onset, or whether it emerges through progressive selection within a chronically IL-15 rich niche (77). To our knowledge, no validated biomarkers predict which patients with RCD II will progress, which is a substantial limitation for clinical surveillance. Aberrant IELs in RCDII expand independently of gluten exposure under the influence of IL-15 and are further supported by dendritic cells and monocyte derived cells, which promote proliferation, inhibit apoptosis and induce IFNγ secretion through cell-cell interaction (58, 61, 77). This establishes a self-amplifying inflammatory loop that sustains innate immune activation independent of classical adaptive TCR signalling (69, 78). The abnormal IELs exhibit selective overexpression of IL-15 receptor βγ chains, making them hypersensitive to low levels of IL-15 and resulting in activation of JAK3/-STAT5 signalling pathway (79).

RCDII is now viewed as a low grade clonal intraepithelial lymphoproliferation disorder arising from a rare subset of innate-like T-IEL precursors. These cells are characterised by the absence of surface CD3 and TCR expression, retained cytoplasmic CD3 and rearranged TCR genes, expression of NK receptors such as NKp46 and cytotoxicity activity in response to IL-15. During progression, recurrent gain of function mutation in JAK1 or STAT3 further amplifies cytokine responsiveness, allowing aberrant IEL clones to outcompete normal resident T-IELs and accumulate additional genetic alterations that drive transformation into aggressive EATL within 5 years. EATL represents the downstream malignant consequence of persistent innate IEL reprogramming, especially in RCDII (4, 37, 61, 67, 68) (**Figure 3**).

**Figure 3:**
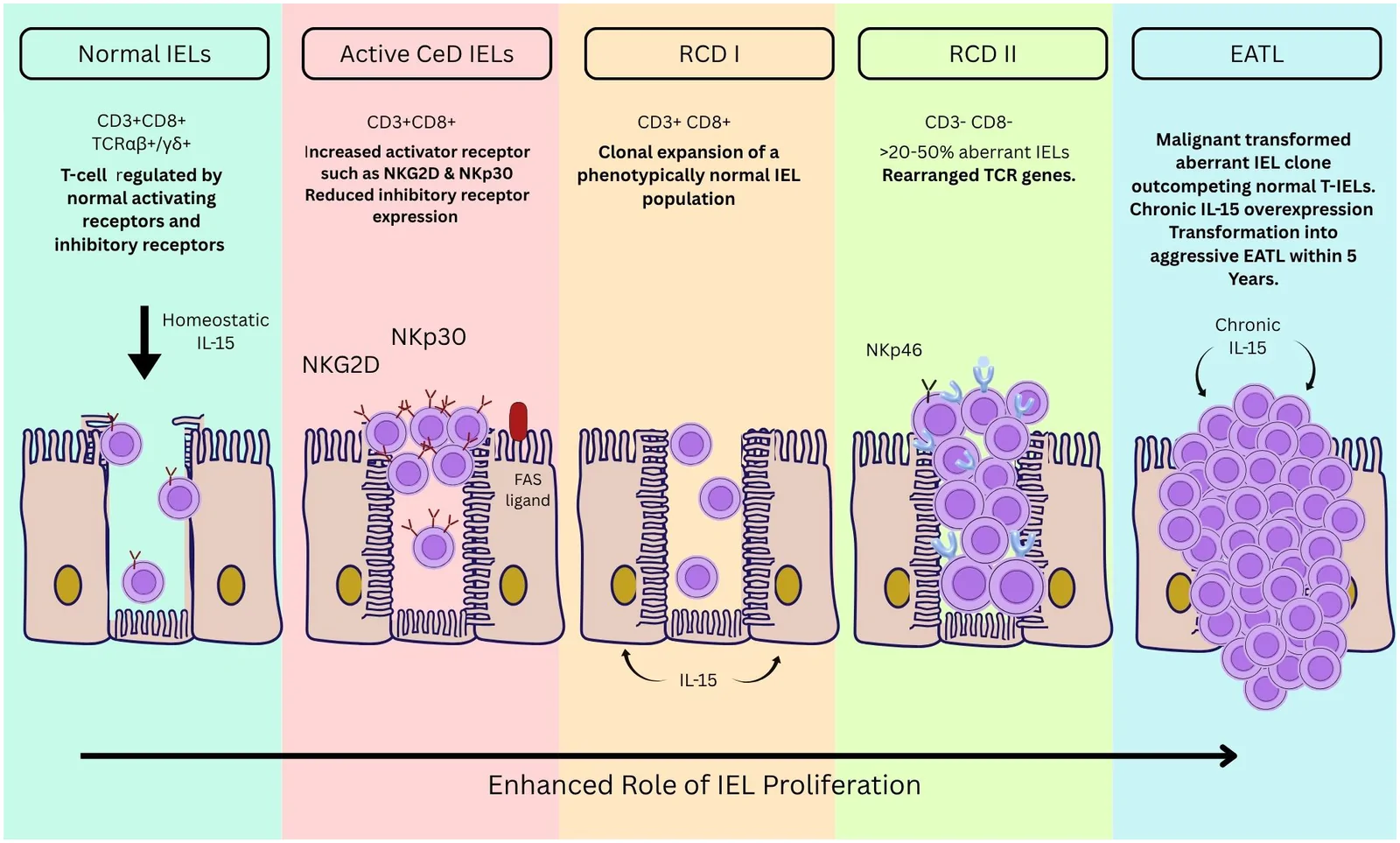
IEL reprogramming in RCD and progression towards EATL. IELs, Intraepithelial lymphocytes; CeD, Celiac Disease; RCD, Refractory Celiac Disease; TCR, T-cell receptors; GFD, Gluten free diet; EATL, Enteropathy associated T-cell lymphoma.

Previously, EATL was classified as EATL type I and type II, preceded by RCD. In 2016, the terminologies were revised by WHO. EATL type I is now termed as EATL and represent lymphoma associated with celiac disease, whereas EATL type II is termed as monomorphic epitheliotropic intestinal T-cell lymphoma (MEITL). MEITL has a broader distribution and occurs in areas where celiac disease is rare. It’s a *de novo* entity often seen in Asian population, whereas EATL is more common in northern Europe and Netherlands (80). EATL and MEITL differ immunophenotypically and molecularly. EATL typically shows a TCR-silent phenotype and frequent JAK/STAT pathway alteration, whereas MEITL expresses CD56 and carries SETD2, STAT5B, JAK3, TP53 and GNAI2 alterations (81). According to ICC, RCD II is now recognised as an *in situ* precursor of EATL because of its clonal aberrant IEL population and shared JAK/STAT abnormalities. RCD II is therefore no longer regarded as only inflammatory complication of CD, but as an early neoplastic condition (82).

Apart from lymphocytes, granulocytic infiltration is a consistent but less studied feature of CD mucosa. Mast cell density increases with lesion severity and correlates with disease progression. Mast cell derived mediators also contributes to epithelial barrier disruption and neutrophil accumulation (83). However, the functional contribution of these populations relative to IL-15 and IEL axis has not been explored yet.

### 2.3 RCD: contributing factors

In addition to the presence of HLA DQ2/DQ8 haplotypes, several other factors contribute to the development of celiac disease including non-HLA genetic factors, epigenetic regulators, gut microbiome dysbiosis, environmental triggers such as viral infection. An overview of the contributing factors is provided in **Figure 4**. Variation in immune regulation also plays a critical role, as some individuals exhibit an exaggerated immune response to gluten despite having similar genetic backgrounds (1).

**Figure 4:**
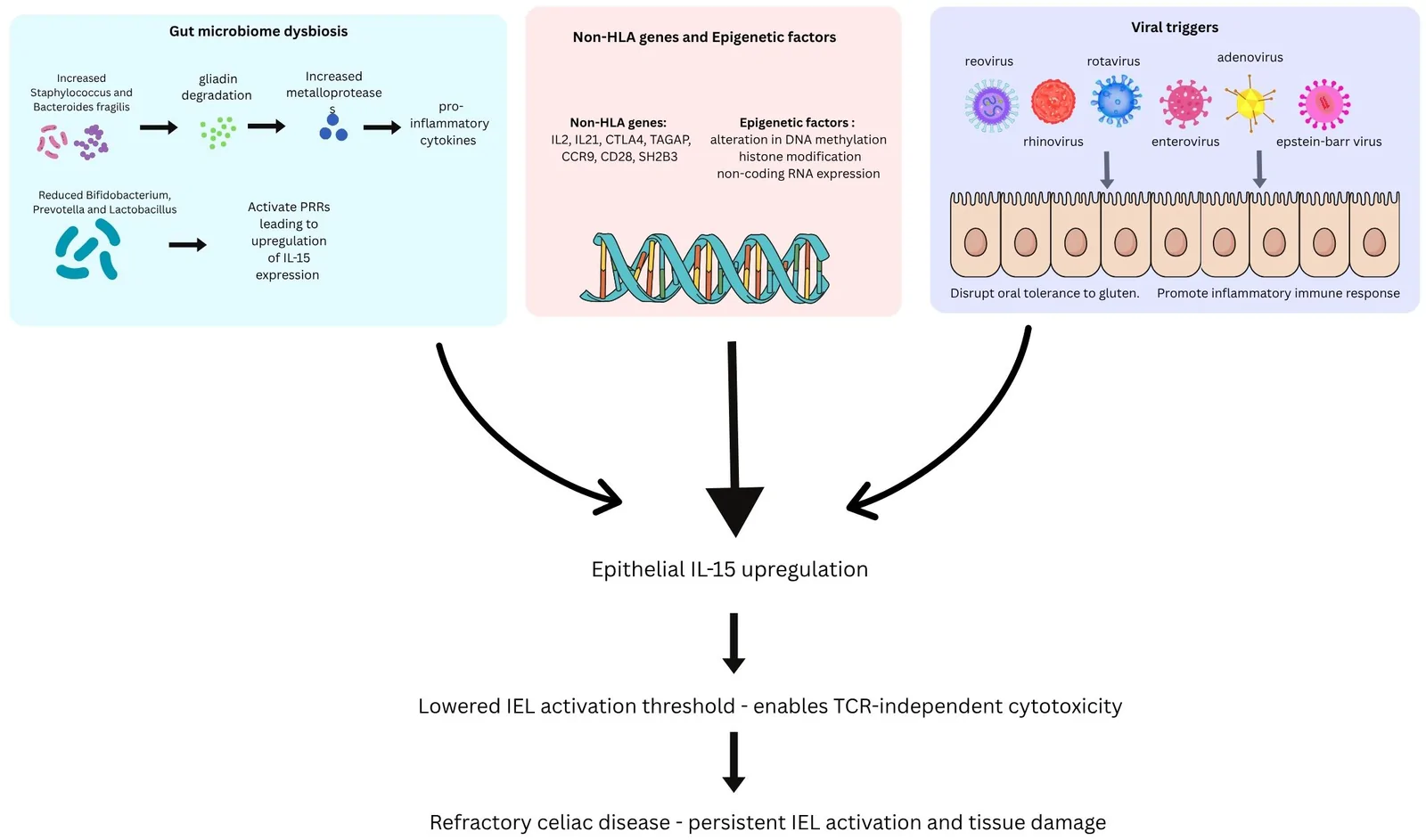
RCD: contributing factors. TLR3, Toll like receptors 3; RIG-I, Retinoic acid-inducible gene I; IELs, Intraepithelial lymphocytes.

#### 2.3.1 Non-HLA genes and epigenetic regulations

As mentioned above, HLA susceptibility is necessary but not sufficient for disease development (84–86). Genome-wide studies have identified more than 50 additional risk variants loci (non-HLA), most of which are located near genes involved in immune regulation within the intestine and thymus (87). These loci can be grouped according to the immune process they affect. THEMIS and RUNX3 acts in T-cell differentiation in thymus. SH2B3, TAGAP, PTPN2, CD28, CTLA4 and IL2/21 are involved in T-cell activation. CTLA4 delivers the opposing co-inhibitory signals whereas CD28 provides the co-inhibitory signals during antigen presentation. RGS1 and CC chemokine receptors govern effector T-cell migration. IL12A, IL18RAP and STAT4 helps in CD4 T-cell differentiation. CCR9 which directs lymphocyte of small intestine, has also been associated with CD. These loci influence the threshold, duration and location of the lymphocyte response. Most of them are present in non-coding regulatory regions and do not alter protein sequence. Many are also shared with other autoimmune conditions. This indicates that they confer general susceptibility to loss of tolerance and the disease specificity is contributed by HLA (88, 89).

Even when combined with HLA, these variants explain only about half of the total genetic risk, suggesting an important role for epigenetic factors. Epigenetic changes in celiac disease include alterations in DNA methylation, histone modifications in both epithelial and immune cells, and these epigenetic signatures may differ between active disease and patient adhering to GFD (85). Methylation patterns differ between cell types. Recent methylation studies have identified a cell type which was hypermethylated. The affected genes mapped to HLA region and included TAP1 and HLA-B. This indicates that HLA contributes to disease through mechanisms additional to DQ allele effect. The overlap between these genes and celiac associated SNPs was minimal, which indicates that methylation changes are more likely to reflect environmental factors (90). Several microRNAs are additionally downregulated in CD, including miR-192-5p, miR-31-5p and miR-338-3p, with corresponding upregulation of their targets FOXP3, RUNX1 and NOD2. These changes can be induced by gliadin exposure which indicates that they responds to antigen (91).

These signatures hold potential not only for improving understanding of disease pathogenesis but also as biomarkers for patient stratification, predict complications, and identification of high risk individuals prior to disease onset. However, several limitations restrict their application. Cohort studies have been small and the methods used to separate cell population can alter methylation pattern. Most of the available data are cross-sectional so it cannot be determined whether the epigenetics changes precede disease or result from the established disease (85).

#### 2.3.2 Gut microbiome dysbiosis

Gut microbiome dysbiosis is strongly associated with celiac disease; however, it remains unclear whether dysbiosis is a cause or a consequence of disease progression. Altered gut microbiota can promote inflammation and disrupt intestinal barrier integrity, allowing harmful substances and microorganisms to enter the body and potentially trigger celiac manifestations (92, 93). Even in patients adhering to a gluten-free diet, the gut microbiota often remains altered compared to healthy individuals, particularly in those with persistent symptoms (94).

Typical findings include an increased abundance of *Staphylococcus* species and *Bacteroides fragilis*, along with a reduction in beneficial bacteria such as *Bifidobacterium* (especially *B. longum*), *Prevotella*, and *Lactobacillus* (92, 93, 95). Certain strains of *Bacteroides fragilis* carry virulence-associated metalloprotease genes, producing enzymes capable of degrading gliadin peptides into immunogenic fragments that stimulate pro-inflammatory cytokine production, including tumour necrosis factor-α (TNF-α), in epithelial cell culture models (92). This is supported by an *in vitro* study in which Caco-2 cells, when exposed to *Bacteroides fragilis* and gliadin, show a subsequent increase in intestinal permeability and increased production of cytokines like TNFα and IL (50, 92). This microbial imbalance is also associated with reduced populations of short-chain fatty acid-producing bacteria, which are important for maintaining intestinal barrier function and limiting inflammation

Microbial metabolism of gluten further influences disease pathogenesis. Bacteria isolated from the duodenum of CD patients, such as opportunistic *Pseudomonas aeruginosa*, generate gluten-derived peptides that effectively cross the intestinal barrier and activate gluten-specific T-cells. In contrast, commensal bacteria such as *Lactobacillus* species degrade gluten peptides into less immunogenic forms, helping to restore microbial dysbiosis, enhance barrier integrity, and reduce inflammatory responses. These findings suggest that microbial processing of gluten modulates antigenic stimulation in genetically predisposed hosts (26, 93).

Early life dysbiosis has also been observed in high-risk HLA DQ2 infants, characterised by reduced *Bifidobacterium*, increased *Firmicutes* and *Proteobacteria* and lower secretory IgA. These alterations may influence innate immune maturation contributing to increased epithelial permeability and enhanced IL-15 responses that can persist into refractory disease (96).

*In vivo* mouse colonisation models, employed to investigate the influence of human duodenal bacterial isolates on gluten metabolism and immunogenicity demonstrated that, mice colonised with bacteria derived from CD patients generated gluten peptide profiles that differed markedly from those observed in mice colonised with bacteria from healthy individuals. The immunogenicity of the resulting peptides was subsequently confirmed using peripheral blood mononuclear cells from patients with celiac disease. These integrative *in vivo* approaches provide compelling evidence that gut microbiota composition can directly modulate gluten peptide processing and subsequent immune activation, thereby contributing to the inflammatory processes characteristic of celiac disease (26).

Beyond gluten metabolism, dysbiosis contributes to celiac disease through modulation of innate immune signalling pathways. Microbial products can activate pattern recognition receptors, such as TLR4 activation by LPS or MyD88-dependent signalling in epithelial cells, leading to upregulation of IL-15 and IL-15Rα expression. This provides a mechanistic link between microbial imbalance and sustained IL-15 signalling, reinforcing chronic innate immune activation in celiac disease (69).

#### 2.3.3 Viral triggers

Beyond Gluten exposure and genetic susceptibility, viral infections have emerged as an important environmental factors in the progression of RCD (28, 46). Epidemiological and experimental studies suggest that early-life viral infections may disrupt oral tolerance to gluten and promote pro-inflammatory immune responses. Several viruses, including reovirus, rotavirus, rhinovirus, enterovirus, adenovirus, Epstein-Barr virus, norovirus and SARS-CoV-2, have been associated with CD pathogenesis and disease progression (97, 98). In particular, reovirus infection has been shown to induce inflammatory responses to dietary antigens and impaired immune tolerance to gluten through activation of type I interferon responses, which alter dendritic cell function towards pro-inflammatory antigen presentation and promotes loss of tolerance to dietary antigens (9, 99).

At the epithelial level, viral infection activates an innate immune pathway that overlaps with mechanism already dysregulated in celiac disease. Type I and type III interferons enhance epithelial stress responses, increase expression of non-classic MHC molecules and stress ligands and directly upregulate IL-15 production. In addition, viral activation of PRRs, including TLR3 and RIG-I-like receptors, triggers MyD88 and IRF-dependent signalling cascade, further amplifying cytokine release and sustaining a pro-inflammatory epithelial environment (9, 98, 100).

Observations during the COVID-19 period have also highlighted viral-immune interactions in predisposed individuals, including reports of post-infectious gluten sensitivity, delayed mucosal healing and persistent inflammatory symptoms. In RCD, viral infection may function as a secondary trigger that sustains epithelial stress signalling and chronic IL-15-mediated activation of IELs. This may promote progression from reversible gluten-dependent inflammation towards persistent antigen- independent innate immune activation, which is a characteristic of RCD (101).

## 3 RCD: management

### 3.1 Diagnosis

As depicted in **Figure 5**, celiac disease is typically diagnosed through a combination of serological testing and duodenal biopsy. Serological screening is usually performed first, most commonly using IgA anti-tissue transglutaminase antibodies, which are highly sensitive and specific (102).

**Figure 5:**
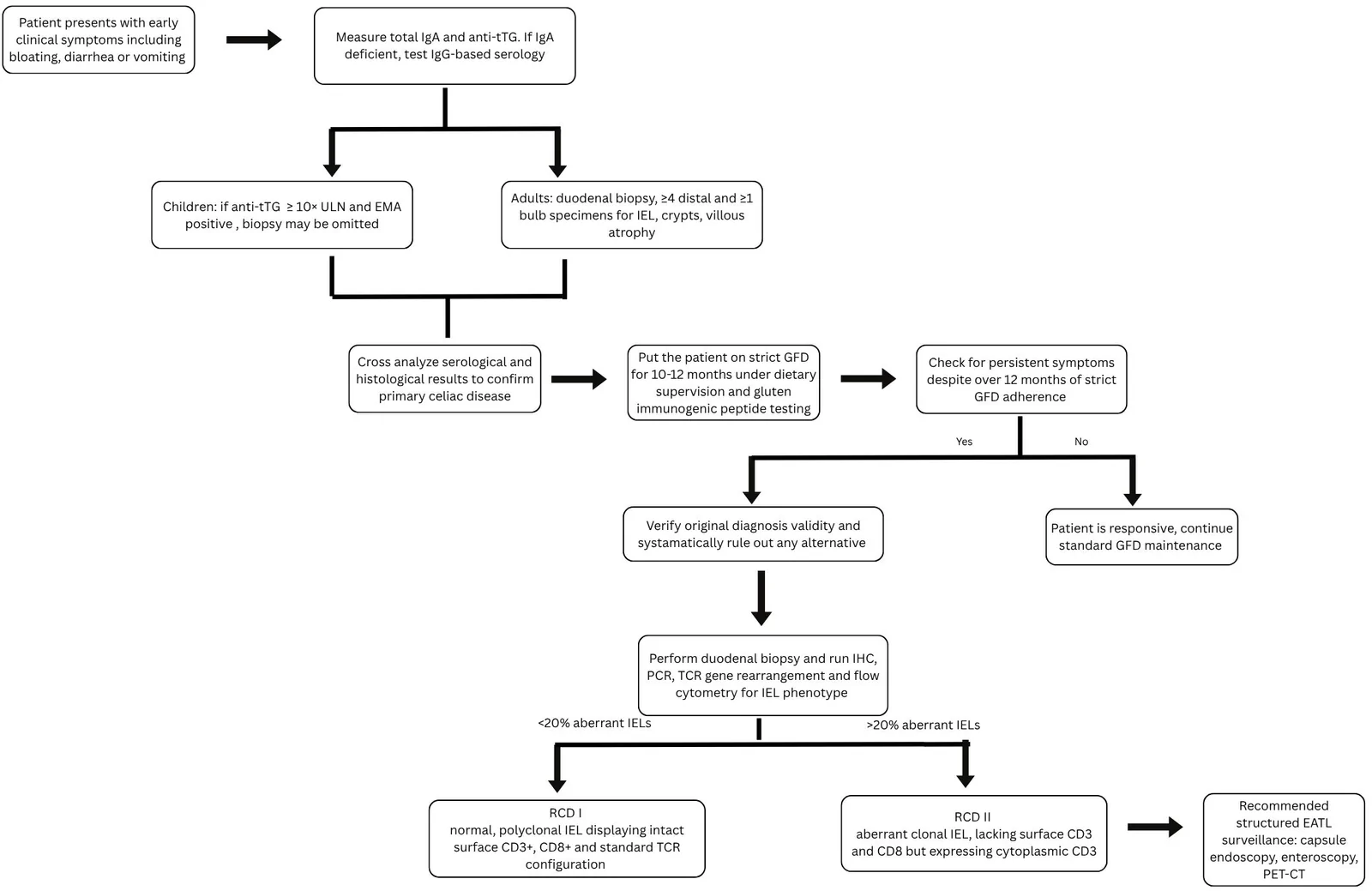
Diagnostic flowchart of RCD. Anti-tTG, Anti Tissue-transglutaminase; GFD, Gluten free diet; RCD I, refractory Celiac Disease type I; RCD II, Refractory Celiac Disease type II; IHC, Immunohistochemistry; EATL, Enteropathy Associated T-cell Lymphoma.

The histological findings include increased intraepithelial lymphocytes, crypt hyperplasia and villous atrophy. These are graded using the Marsh-Oberhuber classification, which recognises five categories of lesion. Type 0 is a preinfiltrative lesion with normal mucosa. Type 1 is an infiltrative lesion, with increased IELs. Type 2 is infiltrative and hyperplastic with crypt hyperplasia added to lymphocytosis. Type 3 is the flat destructive lesion, and is divided into 3a with partial villous atrophy, 3b with subtotal atrophy and 3c with total atrophy. Type 4 is an atrophic and hypoplastic lesion (103, 104).

However, the Marsh-Oberhuber system has recognised limitations. Simplified systems such as the Corazza-Villanacci classification have been proposed to improve reproducibility (104). Histological interpretation is further complicated by the fact that intraepithelial lymphocytosis is not specific to CD and similar appearances occur in *H.pylori* infection, small intestinal bacterial overgrowth and Olmesartan associated enteropathy.

Immunohistochemistry adds information that routine haematoxylin and eosin staining cannot provide, and it becomes essential when RCD is suspected. In normal small intestine, IELs express CD3, CD8, CD103 and TCR β, and they also express the inhibitory receptor LAIR1. An increased proportion of TCRγδ+ IELs in characteristic of CD, and because this elevation persist after mucosal healing it retains diagnostic value in patient who have already started a GFD (65).

Patients who fail to improve despite strict adherence to GFD should be reassessed for refractory celiac disease (RCD) (27). Diagnosis of RCD follows a stepwise exclusion process. It requires confirmation of the original diagnosis of celiac disease, negative celiac serology, strict adherence to GFD confirmed by an expert dietitian and exclusion of other causes of villous atrophy. Duodenal biopsies are performed to confirm persistent mucosal damage, and additional investigations such as capsule endoscopy or device-assisted enteroscopy may be used to identify complications (16, 105).

RCD type I is characterised by polyclonal, phenotypically normal IELs expressing surface CD3+, CD8+, T-cell receptor (TCR). This can be assessed using flow cytometry, immunohistochemistry and PCR-based T-cell receptor analysis, which demonstrates a polyclonal TCR repertoire (102). In contrast, RCD type II is defined by the presence of a clonal population of aberrant IELs lacking surface CD3 and CD8 but expressing cytoplasmic CD3. Diagnosis relies on a combination of flow cytometry, immunohistochemistry and TCR gene rearrangement analysis. Flow cytometry is preferred when available due to its higher sensitivity and ability to distinguish surface from cytoplasmic CD3 expression (37, 106). Flow cytometry is considered the gold standard for identifying aberrant IEL populations in RCDII due to precise surface vs cytoplasmic CD3 expression (107). It enables accurate differentiation between RCD subtypes (102). Fresh biopsy samples preserved in saline or RPMI medium are required for optimal analysis (36).

An aberrant IELs population exceeding 20-50% of total IELs supports the diagnosis of RCD type II. However, IEL clonality alone is insufficient for diagnosis, as clonal populations may also be present in uncomplicated celiac disease. Therefore, diagnosis must integrate clinical presentation, histology, IEL immunophenotype and molecular findings (68).

Metabolomic and microbiota-based studies suggest that CD is associated with alterations in energy metabolism, amino acid processing, and microbially derived metabolites. These findings highlight their potential as non-invasive biomarkers complementary to serology and histology. However, variability across patient populations, dietary patterns and analytical methods currently limits their application in routine diagnosis or in distinguishing refractory from responsive disease (108).

### 3.2 Therapeutic strategies in RCD

The growing understanding of innate immune dysregulation in RCD has led to the development of many mechanisms based therapeutic strategies. These approaches are broadly divided into two categories: therapies targeting gluten and initial triggers and therapies targeting inflammation and disease progression. Therapies targeting gluten and initial triggers include dietary interventions, passive oral immunity, enzyme-based therapies and CRISPR/Cas9 gene editing whereas therapies targeting inflammation and disease progression include probiotic and microbiota modulation, nutraceuticals, IL-15 pathway, immunosuppressants and stem cell therapy. All therapeutic strategies are presented in **Figure 6**.

**Figure 6:**
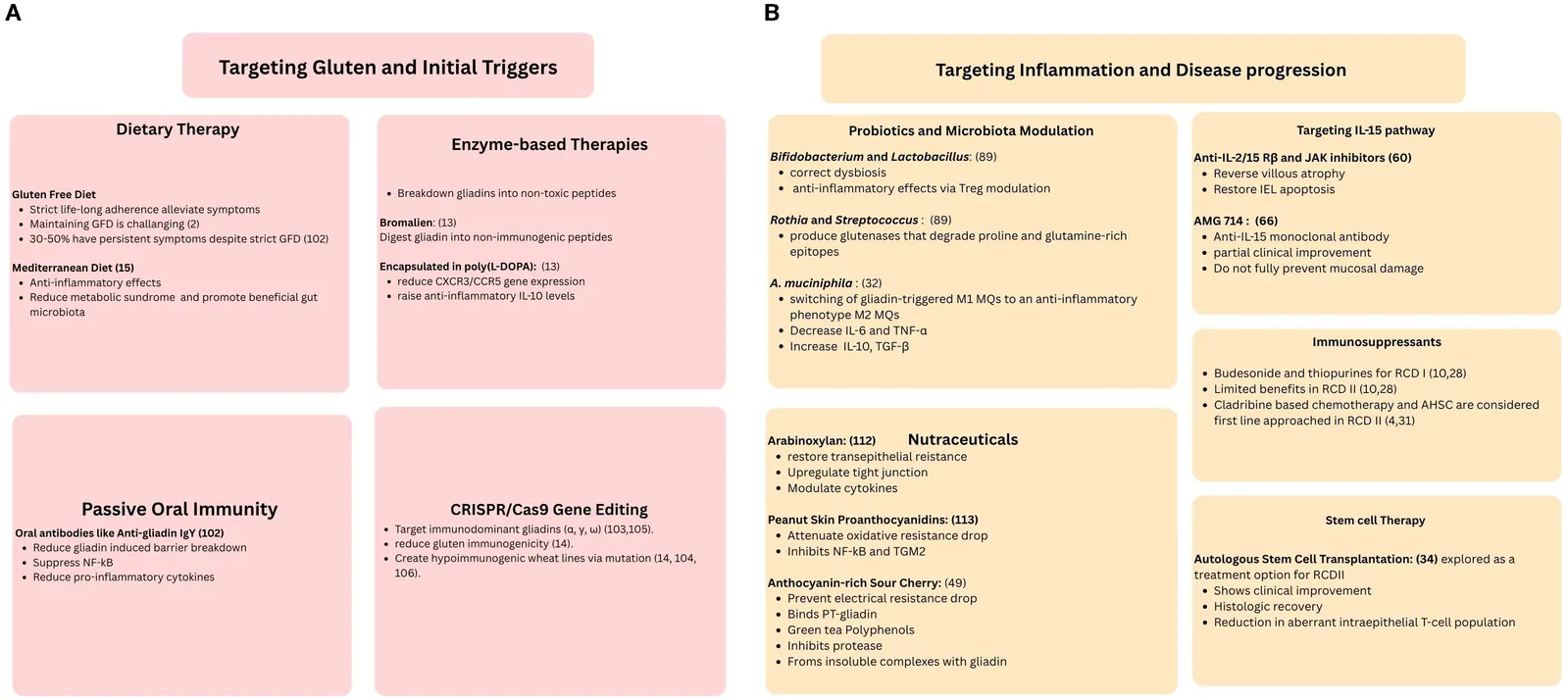
**(A)** Therapies targeting gluten and initial triggers. GFD, Gluten Free Diet. **(B)** Therapies targeting inflammation and disease progression. RCD I/II, Refractory Celiac Disease type I/II; AHSC, Autologous Haematopoietic Stem Cell.

#### 3.2.1 Targeting gluten and initial triggers

##### 3.2.1.1 Dietary therapy for RCD

The standard treatment for celiac disease is strict lifelong adherence to a gluten-free diet, which alleviates symptoms, heals the mucosal lining, normalises serological markers, and reduces the risk of long-term complications (2, 19, 34). However, maintaining a gluten-free diet can be challenging due to the widespread presence of gluten in food and consumer products (2, 19). Despite strict adherence, approximately 30-50% of patients continue to experience gastrointestinal symptoms and persistent intestinal damage. In addition, a small subset of patients (approximately 1%) shows no clinical improvement with dietary modification, a condition known as refractory celiac disease (109). These limitations highlight the need for alternative therapeutic strategies beyond dietary restriction.

The Mediterranean diet (MD) has well-established anti-inflammatory effects. Adherence to this dietary pattern has been associated with a reduced incidence of obesity and metabolic syndrome, as well as decreased mortality and morbidity in patients with cardiovascular diseases. The traditional MD is characterised by a high intake of polyphenol-rich foods such as extra-virgin olive oil and red wine, along with vegetables, grains, legumes, wholegrain cereals and nuts. It also features a favourable fatty acid with high levels of monounsaturated and polyunsaturated fatty acids and low levels of saturated fats, along with reduced consumption of processed meat and refined sugars. Emerging experimental and clinical evidence suggests that the MD promotes a beneficial gut microbiota profile. It enhances microbial diversity and activity, thereby improving host metabolic function. In conclusion, dietary patterns can affect inflammation and disease processes through multiple interconnected mechanisms (21).

##### 3.2.1.2 Passive oral immunity

An *in vitro* study shows that the application of specific anti-gliadin Ig Y antibodies significantly reduced gliadin-induced barrier breakdown, suppressed NF- κB phosphorylation, and decreased IL-8 secretion (2). In another study, the researchers used Caco-2 monolayers exposed to PT-gliadin to mimic gliadin-induced epithelial injury. Experimental conditions included controls (PBS, PD-casein), PT-gliadin alone, PT-gliadin with non-specific IgY, and PT-gliadin with anti-wheat gliadin IgY antibody at various ratios. Integrity of the epithelial layer, gliadin absorption, and production of pro-inflammatory cytokines were assessed. The anti-gliadin IgY at a 1:3,000 ratio significantly prevented gliadin-induced loss of epithelial integrity, reduced phenol red permeation, and decreased pro-inflammatory cytokine production compared with PT-gliadin alone (110).

##### 3.2.1.3 Emerging enzyme-based therapies

Enzyme-based therapies show considerable promise in this context. They break down gliadin proteins into peptides, which are nontoxic and have anti-inflammatory effects. The mechanism underlying this therapeutic strategy is that most gliadin-degrading enzymes cleave at proline and glutamine residue sites, thereby breaking down gluten proteins into smaller fragments. This effectively neutralises the toxicity of gliadin (19).

Bromelain is a cysteine protease derived from pineapple. It has broad specificity for hydrophobic residues and anti-inflammatory properties, and effectively digests gliadin into non-immunogenic peptides. Encapsulated in novel poly(L-DOPA)-coated silica nanocomposites, bromelain retained full activity without cytotoxicity in Caco-2 cells. It also reduced CXCR3/CCR5 gene expression in gliadin-exposed enterocytes. In celiac patients, it reduces pro-inflammatory cytokines and raises anti-inflammatory IL-10 levels. It worked better than free bromelain, which confirms that the coating preserved the immunomodulatory effect, making it a reliable treatment option where GFD fails (19).

##### 3.2.1.4 CRISPR/Cas9 mediated gene editing

CRISPR/Cas9 gene editing targets immunodominant gliadins (α, γ, ω) in wheat. These gliadins encode the most abundant CD epitopes DQ 2/DQ8, reducing gluten immunogenicity whilst preserving HMW/LMW glutenin baking functionality (20, 111–113). Few studies successfully edited multiple gliadin loci simultaneously, creating hypoimmunogenic lines with 10-90% epitope reduction via targeted deletion or inactivating mutation (20, 112, 114). Combined with RNAi silencing and classical breeding of natural epitope null variants, this approach offers a promising adjunctive strategy to strict GFD for n RCD patients by minimising inadvertent gluten exposure triggers (111, 115).

#### 3.2.2 Targeting inflammation and disease progression

##### 3.2.2.1 Probiotics and microbiota modulation

Another potential treatment option for celiac disease can be the use of probiotics (16). As gut microbiome dysbiosis affects the disease condition, correcting this dysbiosis using probiotics like *Bifidobacterium* and *Lactobacillus* can help in reducing the symptoms of the disease (93, 95).

Oral or duodenal microbiota like *Rothia and Streptococcus* produce glutenases that degrade proline and glutamine-rich epitopes, whereas *Lactobacillus helveticus* and *Bifidobacterium bifidum* exhibit anti-inflammatory effects via Treg modulation and FOXP3 splicing. Probiotic trials show improved symptoms and microbiota balance in CD patients on GFD (95). Emerging glutenases include ALV003 (barley EP-B2 + *S.capsulata* PEP), which reduced villous atrophy during 2g gluten challenge (2 grams of gluten daily), and AN-PEP (*A.niger* PEP), which detoxifies 8g gluten (50, 116). These results support the potential of passive oral antibody-based approaches as adjunctive or alternative strategies to strict gluten avoidance in the management of celiac disease (95, 110).

The positive effects of *A. muciniphila*, on phenotype switching of gliadin-triggered M1 MQs to an anti-inflammatory phenotype were also observed in this study. In our model, pre-treatment of gliadin-stimulated MQs with *A. muciniphila* (evaluating the preventive effect) caused increased M2 (CD206 +) phenotype MQ and reduced M1/M2 MQ ratio. This transition from M1 to M2 phenotype was associated with decreased proinflammatory (IL-6, TNF-α) and increased anti-inflammatory (IL-10, TGF-β) cytokines expression (31). It is worth mentioning that *A. muciniphila* has mucin-degrading enzymes and utilises mucin, a complex glycosylated protein, and degrades it as carbon, energy and nitrogen sources (38). These beneficial by-products are involved in regulating the host immune system through different signals, including TNF‐α, IL‐10, etc. (117).

##### 3.2.2.2 Nutraceuticals based approaches

Arabinoxylan, a hexose-based plant-derived polysaccharide, has demonstrated protective effects on intestinal epithelial barrier integrity. Arabinoxylan hydrolysate (AXX), characterised by a high degree of substitution and molecular weight, restores transepithelial resistance in Caco-2 cells following LPS-induced permeability. It upregulates tight junction proteins, including zonulin, occludin and claudin-1 and modulates cytokine profiles by increasing IL-2 whilst reducing pro-inflammatory cytokines such as IL-6, IL-10 and TNFα. AXX inhibits the MyD88/NF-κB signalling pathway and activates protein kinase C-mediated barrier protective pathways. In addition, it promotes growth of beneficial microbiota, including Bifidobacterium and *Lactobacillus* species, and enhances short-chain fatty acid production, such as acetate, propionate and butyrate (118).

Peanut skin proanthocyanidins (PSPc) have shown the ability to attenuate gliadin-induced oxidative stress and inflammation. PSPc enhanced antioxidant defence mechanisms through activation of the SIRT1/NRF2 signalling pathway whilst suppressing inflammatory signalling via SIRT1- and IκB-dependent inhibition of NF-κB activation. This results in reduced secretion of pro-inflammatory cytokines, including IL-1β, IL-6, and TNF-α. Additionally, PSPc improved epithelial cell viability, preserved monolayer integrity, and reduced the expression of transglutaminase 2 (TGM2), a key mediator of gliadin-induced epithelial damage (119).

Anthocyanin-rich sour cherry extract, containing cyanidin-3-O-glucosylrutinoside, has demonstrated the ability to bind pepsin-trypsin-digested gliadin at intestinal pH. Pretreatment prevents the reduction in transepithelial electrical resistance caused by chronic gliadin exposure and restores the normal localisation of tight junction proteins. It also inhibits NF-κB p65 nuclear translocation and significantly reduces the secretion of pro-inflammatory cytokines, including TNF-α, IFN-γ, and IL-8, thereby improving epithelial barrier integrity (54).

Emerging evidence also supports luminal gliadin sequestration as a potential therapeutic strategy. Green tea polyphenols can inhibit protease activity in a dose-dependent manner and form insoluble complexes with gliadin peptides, thereby reducing their digestion, epithelial translocation and subsequent inflammatory responses, including IL6 and IL8 production. This approach may help prevent sustained innate immune activation by limiting antigenic stimulation that drives IL-15 overexpression and IEL cytotoxicity in refractory celiac disease (120).

##### 3.2.2.3 Targeting IL-15 and downstream pathway

The central role of IL-15 in driving cytotoxic IELs activation and sustaining aberrant IEL survival in RCD II has led to the development of IL-15 targeted therapies, including anti IL-2/15 Rβ antibodies and JAK inhibitors. Preclinical studies have demonstrated that targeting IL-15 signalling can reverse villous atrophy and restore IEL apoptosis, highlighting its therapeutic potential (67). Targeting persistent cytokine signalling, therefore, represents a therapeutic promise for refractory disease (4).

In a rhesus macaque model, the anti-IL-15 antibody 04H04 showed encouraging results, including a reduction in IEL numbers, CD8+IFN-γ+ and CD4+IFN-γ+ T cells, and circulating NK cells. It also improved the villus-to-crypt ratio and intestinal architecture, even in the presence of dietary gluten. These findings suggest that combining IL-15 blockade with dietary control may offer enhanced long-term benefits in refractory celiac disease (76, 116).

Early clinical trials with anti-IL-15 monoclonal antibody AMG 714 have demonstrated both potential and limitations. In RCDII patients, treatment resulted in partial clinical improvement, including reduction in diarrhoea and improvement in IEL TCR clonality. However, it did not fully prevent ongoing mucosal damage. These findings indicate that IL-15 blockade alone may be insufficient, and that combination strategies or targeting shared downstream pathways such as JAK/STAT signalling may be required for effective disease control (73).

##### 3.2.2.4 Immunosuppressant

Current guidelines recommend oral budesonide as first-line therapy for RCDI, with thiopurines as a second-line immunosuppressive agent. Conventional immunosuppressive therapies, including corticosteroids and broad-spectrum agents such as azathioprine and cyclosporin, have shown limited benefit in refractory celiac disease, particularly in type-II disease, where IELs exhibit autonomous survival advantages despite dietary gluten exclusion (16, 34). Cladribine-based chemotherapy and autologous haematopoietic stem cell transplantation are generally considered first-line approaches due to the high risk of progression to EATL (3, 4, 37).

##### 3.2.2.5 Stem cell-based therapy

RCD II is usually resistant to many known therapies, including azathioprine, prednisone, cyclosporin and IL-10 therapy and has a very high risk of developing EATL. In this situation, autologous stem cell transplantation (ASCT) has been explored as a treatment option. In small pilot studies, high doses of chemotherapy followed by ASCT were found to be effective and resulted in clinical improvement, histologic recovery and a significant reduction in aberrant intraepithelial T-cell population (40).

## 4 Discussion

The evidences reviewed above supports a model in which celiac disease begins as an adaptive, gluten specific response but transition into a state maintained by innate mechanisms. Distinct NK and NKT cell signatures associated with active celiac disease highlights the importance of innate immunity in disease pathogenesis (28). Alterations in vesicular trafficking amplify gliadin-induced stress and inflammatory responses, and these changes acts before adaptive immunity recognition takes place (31). Hence, innate model explains why the damage continues even without antigen. Once epithelial stress, IL-15 trans presentation and NKG2D mediated killing form a reinforcing circuit, mucosal damage can continue even after gluten is removed. However, the innate model does not explain why only a small number of patients with CD develop RCD when the same circuit is present in all patients with active disease.

IL-15 is a central part of the innate immune circuit and hence several drugs have been developed to target it. However, the clinical results have been modest. In the phase 2 trial of AMG 714 in RCD II, primary endpoint of reduced aberrant IELs was not seen on flow cytometry, although symptoms and some secondary endpoints improved (121). There are possibly three reasons for this. First, aberrant IEL clones in RCD II already carry gain of function mutation in JAK1/STAT3, so their signalling is partly independent of the cytokines hence blocking only IL-15 may not be enough. Second, IL-15 is presented on the cell membrane rather than released freely, which may make it harder for a circulating antibody to reach. Third, IL-21 and other ϒ-chain cytokines act alongside IL-15 and may keep IELs alive when IL-15 is blocked. These points suggest that combination therapy, or targeting the shared JAK/STAT pathway instead of cytokine is more likely to work for therapeutics.

The fundamental reason as to why only some patients progress to RCD II is unknown yet. Aberrant clones in RCD II carry JAK1/STAT3 mutations at diagnosis and the same mutations are found in subsequent lymphoma. This indicates that the transformation begins well before the lymphoma appears (122). Prolonged diagnostic delay may therefore be relevant, as extended exposure to IL-15 provide more opportunity for clonal selection. permanent changes in the resident IEL population may also contribute, as the loss ofCD8αα+ TCRγδ subset is not reversed by gluten withdrawal (64). However, none of these findings has yielded a clinically usable marker. At present, there is no validated method to predict which patients with RCD I will progress to RCD II, or which patient with RCD II will develop lymphoma.

In this context, therapies directed at gluten itself remain the most direct approach for typical celiac disease. Recent advances in genome editing technologies have demonstrated the potential of CRISPR/Cas9–based multiplex editing to selectively reduce or eliminate immunogenic gliadin proteins in wheat. The development of wheat lines with diminished γ- and ω-gliadin content, and potentially in combination with α-gliadin modifications, suggests a promising strategy to substantially lower the immunotoxin gluten load (20). Microbiome based strategies work on the same variable. Targeted manipulation of small intestinal microbial communities such as enhancing the abundance of gluten-detoxifying commensals like *Lactobacillus* species or limiting the presence of potentially pathogenic bacteria such as *Pseudomonas* may help modulate gluten peptide immunogenicity and dampen intestinal inflammation (26). Probiotics, prebiotics, or broader microbiota-modulating approaches, may offer promising adjunctive strategies to reduce gliadin-driven inflammation and improve intestinal barrier function (92). IgY antibodies may act as an adjunct by neutralising gluten peptides directly, although this still needs suitable oral formulation and *in vivo* testing (110). However, all of these act on how much antigen is present, and RCD is independent of antigen. Hence these are more likely to help patients with CD than those who have already developed RCD.

A different set of consideration applies to nutraceuticals. Natural antioxidant and anti-inflammatory compounds have been studied as adjunct therapies, and proanthocyanidins and similar compounds may improve epithelial resistance to gliadin-induced inflammation through SIRT1/NRF2 and NF-κB signalling (119). The recognition of systemic oxidative stress and DNA damage in celiac disease supports the use of antioxidant and oxidative damage markers as biomarkers as indicators of disease activity (52). However, there are few limitations with nutraceutical approaches. To our knowledge, no randomised trials of any nutraceutical have been carried out in RCD. Hence, nutraceutical compounds are promising but need further confirmational studies.

The uncertainties of the disease get more complicated because of the models available. Caco-2 and similar cell lines used commonly do not contain lymphocytes that are central to the disease (50). Animal models reproduce parts of the disease but not all of it. IL-15 transgenic mice expressing HLA-DQ8 show a marked increase in cytotoxic IELs and inflammation, but they do not develop villous atrophy. No animal model reproduces RCD II or progression to EATL (63). The two subtypes of RCD also differ considerably in outcome. In a single centre cohort of 57 patients, the five year cumulative survival was 80% for RCD I and 45% for RCD II. Most of the deaths occurred within first two years after diagnosis of the refractory state. The refractory state itself was the common cause of death in RCD I, whereas EATL was the common cause in RCD II (123). EATL develops in 33-52% of patients with RCD II within five years of diagnosis, and apart from increased age at diagnosis, the factors that predict progression to EATL are not firmly established (34).

The rarity of the disease creates further problem for the research. The phase 2 trial of AMG 714 included only 28 patients in total, which makes it difficult to tell whether a treatment truly does not work or whether the study was simply too small (121). Patients with CD also carry a substantial economic burden, which is likely to be greater in RCD because of the additional need for the specialist follow up. Gluten free versions of cereals, pasta and snacks have been reported to cost around 139% more than the equivalent wheat based products (124). However, the cost of food is only part of the picture. A recent review found that the social burden extends well beyond gluten free food, and includes excess healthcare use for complications and comorbidities as well as reduced work productivity (125).

## 5 Conclusion

Refractory celiac disease is best understood not as a more severe form of celiac disease, but as a different condition in which the innate immune response in the epithelium has become self-sustaining. Chronic IL-15 signalling, stress ligand expression and NKG2D mediated cytotoxicity form a circuit that no longer needs gluten. This has now been reflected in classification, as RCD II is recognised as an early precursor of EATL rather than an inflammatory complication, and its aberrant IELs already carry transforming mutations at diagnosis. The treatment implications follow from this. Blocking IL-15 alone has given only partial benefit, and combination therapy or targeting the shared JAK/STAT pathway is more likely to work. The important need of the hour is a way to predict progression, since no marker currently identifies which patient will progress or transform. Nutraceutical and microbiota-based approaches remain plausible but are supported almost exclusively by *in vitro* cell line data. Further progress will depend on the development of experimental models that incorporate the lymphocyte population central to disease pathogenesis, and on international collaborative networks large enough to run trails in a condition this rare.
